# Low Temperature Stress Mediates the Antioxidants Pool and Chlorophyll Fluorescence in *Vitis vinifera* L. Cultivars

**DOI:** 10.3390/plants10091877

**Published:** 2021-09-10

**Authors:** Mohammad A. Aazami, Majid Asghari-Aruq, Mohammad B. Hassanpouraghdam, Sezai Ercisli, Mojmir Baron, Jiri Sochor

**Affiliations:** 1 Department of Horticulture, Faculty of Agriculture, University of Maragheh, Maragheh 55181-83111, Iran; majid.asghari66@gmail.com (M.A.-A.); hassanpouraghdam@gmail.com (M.B.H.); 2 Department of Horticulture, Faculty of Agriculture, Ataturk University, 25240 Erzurum, Turkey; sercisli@gmail.com; 3 Department of Viticulture and Enology, Faculty of Horticulture, Mendel University in Brno, Valticka 337, 691 44 Lednice, Czech Republic; mojmirbaron@seznam.cz (M.B.); jiri.sochor@mendelu.cz (J.S.)

**Keywords:** biochemical traits, cold stress, gene expression, grapevine, photosynthesis

## Abstract

Grapes are sensitive to early autumn and spring low temperature damage. The current study aimed to assay the effects of cold stress (+1 °C for 4, 8, and 16 h) on three grapevine cultivars (Ghiziluzum, Khalili, and Perllete). The results showed that cold stress caused significant changes in the antioxidant and biochemicals content in the studied cultivars. Furthermore, examining the chlorophyll fluorescence indices, cold stress caused a significant increase in minimal fluorescence (F0), a decrease in maximal fluorescence (Fm), and the maximum photochemical quantum yield of photosystem II (Fv/Fm) in all cultivars. Among the studied cultivars, ‘Perllete’ had the highest increase in proline content and activity of antioxidant enzymes and also had the lowest accumulation of malondialdehyde, hydrogen peroxide, electrolyte leakage, and F0, as well as less of a decrease in Fm and Fv/Fm, and had a higher tolerance to cold stress than ‘Ghiziluzum’ and ‘Khalili’. ‘Perllete’ and ‘Ghiziluzum’ showed reasonable tolerance to the low temperature stress. ‘Khalili’ was sensitive to the stress. The rapid screening of grapevine cultivars in early spring low temperatures is applicable with the assaying of some biomolecules and chlorophyll fluorescence.

## 1. Introduction

Environmental stresses such as cold, salinity and, drought are the most critical factors affecting the growth and productivity of crops. Given the growing population of the planet and the need for more food, producing plants with a high tolerance to environmental stresses is of great importance. One of the factors that limit a plant’s survival and growth is cold stress, which plays an essential role in the ecological distribution of all plants [1]. In adaptation to cold stress, living organisms, predominantly plants, develop several molecular, biochemical, and physiological mechanisms to maintain their survival [2]. Cold stress is a direct result of low temperatures on cellular macromolecules that lead to a slowed metabolism and the loss of membranes function [3]. The cell membrane is the outer living part of a plant cell. When plant membranes are exposed to low temperatures, the membrane’s physical condition changes from a liquid phase to a gel phase that interferes with the membrane dynamics and function. The plasma membrane is a highly organized system that plays an essential role in the relationship between the cell and extracellular matrix. In general, cold stress leads to the loss of membrane health and leakage of solutes [4].

By imposing pressure on the cell wall, a low temperature causes the production of some metabolites, resulting in membrane stability against cold damage and ultimately adaptation to the cold [4]. Cold stress causes changes in cell membrane lipid composition, the amount and function of enzymes, and the accumulation of carbohydrates, amino acids, and soluble proteins in the cell [5]. The other consequence of low-temperature stress is the production of reactive oxygen species (ROSs). ROSs are toxic molecules capable of reacting with and damaging vital molecules such as proteins, nucleic acids, lipids, and carbohydrates. One of the most basic mechanisms for gaining tolerance to the environmental stresses is the elimination of reactive oxygen species (ROSs) [6]. Both enzymatic and non-enzymatic systems are effective in this process to deal with the reactive oxygen species [3,4]. Vineyards are affected by non-living stresses such as cold, drought, salinity, extreme temperatures, chemical toxicity and oxidative stress [1]. Although, the molecular basis of chilling acclimation is poorly understood, the effect of some transcription factors involved in response to low temperature is well-established [7]. 

The photosynthetic apparatus is damaged by cold stress especially in cold sensitive plants. Cold temperatures affect the diverse parts of photosynthesis system, i.e., the stomata’s pore diameter, photosynthetic pigments biosynthesis, the activity of photosystems I and II, the Calvin cycle related enzymatic activity, and CO_2_ acquisition and fixation; hence, the entire photosynthetic capacity and efficiency of plants [8]. Low temperatures and the reduced substituting rate of protein D1 interferes the photosystem II recovery and hence, hugely reduces the related quantum efficacy. Chlorophyll fluorescence is a method of using excitable energy via photosynthesis and is extensively used as a feasible non-destructive procedure for photosynthetic studies in plants, as well as a method broadly employed in the characterization and identification of plant tolerance to the environmental stress factors [9].

Grapevines have huge economic values as fresh fruits, dried products and for winemaking. Climatic variations beside morphological damages greatly impact the quality and composition of vine berries. Low temperatures induce the secondary oxidative stress and hasten the cell metabolic behaviors. Yield losses of up to 90% have been reported in response to early spring low temperatures. Below optimum temperatures influence the plant survival, cell division, photosynthesis, water transport, growth potential and finally the yield and quality of plants. Furthermore, low temperatures modify the photosynthesis normal rhythm and substantially damage cell membranes, leading to reduced CO_2_ acquisition, mainly due to the diminished enzymatic activities [1,3]. 

‘Khalili’, ‘Ghiziluzum’, and ‘Perllete’ are of the major grapevine cultivars in the Iranian agriculture, mainly as fresh fruits (Table grapes). These cultivars are selected based on the preliminary studies conducted on 20 cultivars. The idea was to conduct supplementary evaluations for the efficient and prompt selection of potent cultivars. Applying cold stress, one can select grape cultivars that are more likely to tolerate the low-temperature conditions by monitoring the enzymatic and biochemical activities. The present study aimed to investigate the physiological and biochemical responses of three grape cultivars and their tolerance to cold stress. 

## 2. Materials and Methods

### 2.1. Plant Materials

To investigate the effects of cold stress on three grapevine cultivars, ‘Khalili’, ‘Ghiziluzum’, and ‘Perllete’ their rooted cuttings were transferred to 5 L plastic pots containing one-third each of normal soil, perlite, and blown sand. These cultivars are commonly cultivated in Iran. The homogeneous plant material (2-year-old rooted cuttings) was acquired from the nursery collection of the University of Maragheh, Maragheh, Iran. The plants were nourished with Hoagland’s solution and pH was adjusted to 6.5. This experiment was conducted in the greenhouse of the Department of Horticultural Sciences, University of Maragheh, Iran, as a factorial based on CRD design with three grape cultivars at +1 °C for 4, 8, and 16 h and at 22 °C for the control treatment. With the comparative literature review, there was evidence that the majority of low-temperature stress responsive bio-molecules were more active and functional during the early stress times. Then after, with the prolonged stress-time-course, their amounts remained constant or even declined. We decided to limit the exposure time up to 16 h [7,10,11].

The leaves were sampled after the completion of cold stress treatments to assay the proline, total soluble protein, malondialdehyde, hydrogen peroxide and the activity of the antioxidant enzymes; catalase (CAT), guaiacol peroxidase (GPX) and ascorbate peroxidase (APX). Leaf samples were incubated in liquid nitrogen and kept in the freezer (−80 °C, Jisico Laboratory freezer, Korea) until measurement.

### 2.2. Chlorophyll Fluorescence Indices

Chlorophyll fluorescence was measured by a fluorometer (model: PAM 2500-WALZ, Germany) from the last fifth of the leaves in the light. Minimal fluorescence (F0), maximal fluorescence (Fm), and maximum photochemical quantum yield of photosystem II (Fv/Fm) were assayed.

### 2.3. Proline Content

The proline content was measured in wet plant tissue through the method used by Bates et al. [12] and the absorbance of the samples was recorded at 520 nm wavelength using a spectrophotometer. The control solution contained pure toluene. 

### 2.4. Hydrogen Peroxide

A total of 0.2 g of plant material was homogenized in 2 mL of 0.1% tricloroacetic acid and centrifuged at 12,000× *g* for 15 min. Then 0.5 mL of supernatant was added to 0.5 mL of phosphate buffer (10 mmol, pH = 7) and, 1 mL of iodide potassium (1 mol). The sample’s absorbance was measured at 390 nm. Standard curves were established with the different concentrations of hydrogen peroxide.

### 2.5. Malondialdehyde

A total of 0.2 g of plant sample was homogenized in 2 mL of 20% tricloroacetic acid containing 0.05% TBA. The samples later were incubated at 95 °C for 30 min and were transferred on to ice. The samples were then centrifuged (Hermle Labortechnik, GmbH, Germany) at 10,000 rpm for 10 min and the absorbance was measured at 532 and 600 nm (UV-Vis Spectrophotometer Model 1900i, Shimadzu, Japan). The lipid peroxidation range was obtained from the difference between the absorption wavelengths in the darkness coefficient of 155 mmol cm^−1^.

### 2.6. Total Antioxidant Capacity

The antioxidant capacity of the extracts was calculated as the inhibition percentage of DPPH using the method of Chiou et al. [13].

### 2.7. Antioxidant Enzymes Assay

For the extraction of guaiacol peroxidase (GPX) and soluble proteins, 0.2 g of the sample was homogenized in liquid nitrogen. A total of 2 mL of phosphate buffer (pH = 7.5) containing, EDTA (0.5 mol) was added. The samples were incubated at 4 °C for 15 min and were centrifuged at 15 rpm. Due to the instability and very low half-life of ascorbate peroxidase with ex vivo conditions, and to maintain the structure of the compound, we tried to use polyvinylpyrrolidone 5% and ascorbate (2 mL) to the respected enzyme solution.

#### 2.7.1. Guaiacol Peroxidase (GPX)

For GPX activity, the reaction mixture contained 1 mL phosphate buffer (100 mmol, pH = 7) along with EDTA (0.1 mmol), 1 mL guaiacol (15 mmol), 1 mL H_2_O_2_ (3 mmol) and 50 μL of the extracted enzyme solution. The reaction response was measured at 470 nm for 1 min. Enzymatic activity, based on the amount of tetraguaiacol, was obtained using a darkness coefficient of 26/6 mμ cm^−1^.

#### 2.7.2. Ascorbate Peroxidase (APX)

The APX was assayed as the following: the reaction mixture was contained 250 μL phosphate buffer (pH = 7) along with EDTA, 10 μL H_2_O_2_ (1 mmol), 250 μL sodium ascorbate (0.25 mmol) and 50 μL enzyme solution. The absorbance was measured at 290 nm for 1 min. Enzymatic activity was calculated using the darkness coefficient of 2.8 mmol^−1^ cm^−1^. The resulting number indicates the activity of ascorbate peroxidase based on micromoles of oxidized ascorbate per minute.

### 2.8. Total Soluble Protein Content

The reaction solution contained 100 μL of enzyme solution, 200 μL of Bradford regent and 700 μL of deionizer water. After 2 min of the complex formation, the Bradford regent showed the highest integration with the amino acids. Absorbance was evaluated at 535 nm. Protein content of the samples was calculated based on the standard curve obtained from the defined amounts of bovine serum albumin.

### 2.9. Statistical Analysis

The present study was conducted based on a factorial experiment in a completely randomized design. The data analysis was conducted by the SAS software (version 9.1.3) and means were compared using Duncan’s multiple range test at 5 and 1% probability levels. Tables and graphs were drawn using the Microsoft Office software.

## 3. Results 

### 3.1. Physiological and Biochemical Traits

**Figure 1:**
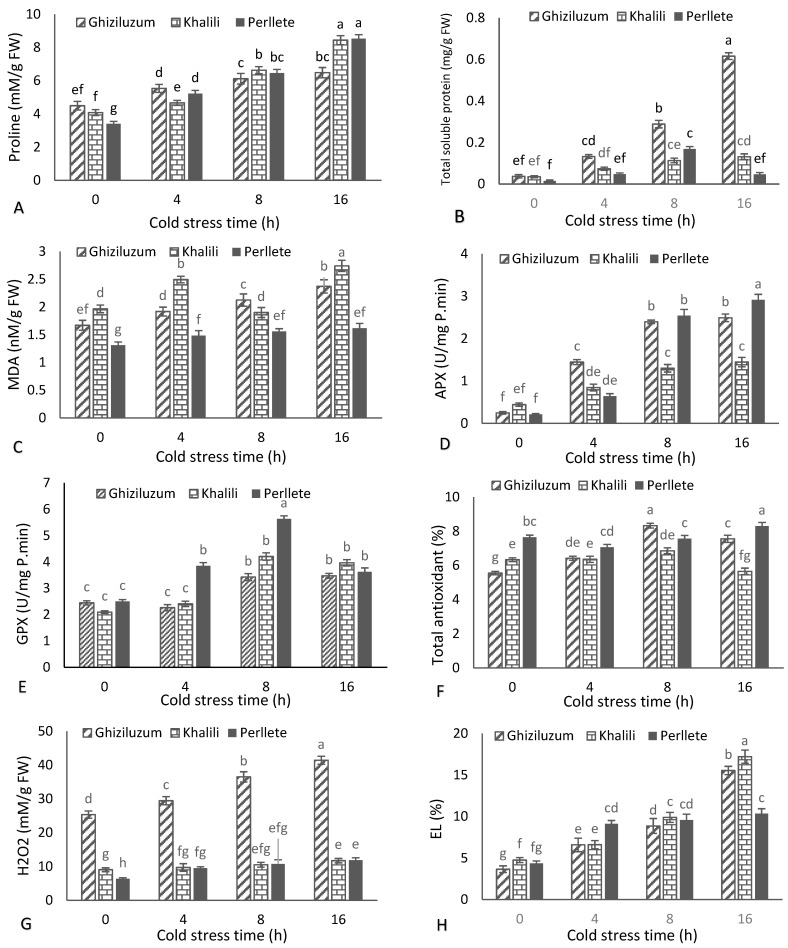
The effects of cold stress time-course on the proline content (**A**), total soluble protein (**B**), malondialdehyde (MDA) (**C**), ascorbate peroxidase (APX) (**D**), guaiacol peroxidase (GPX) (**E**), total antioxidants (**F**), H_2_O_2_ (**G**) and electrolyte leakage (EL) (**H**) of three grapevine cultivars. Similar letters show no meaningful difference at 5% probability level by Duncan’s Multiple Range Test. Data are mean ± SD (*n* = 3).

Considering the interaction effect of stress × cultivar (Figure 1A), cold stress significantly increased proline accumulation in all grapevine cultivars compared with control, and increasing the stress duration led to more proline accumulation in all treatments. In all cultivars, the lowest amount of proline belonged to the control treatment and the highest belonged to 16 h. The most proline accumulation was in the ‘Perllete’ cultivar (8.52 mmol/g of fresh leaf weight) with 16 h of stress application and the lowest proline accumulation was traced in the control treatment of ‘Perllete’ (3.39 mmol/g of fresh leaf weight). The strongest effect of cold stress was on ‘Perllete’ which increased proline accumulation more than the control treatment and the lowest effect belonged to ‘Ghiziluzum’. Mean comparisons (Figure 1B) showed that cold stress significantly changed the total protein content of the leaves, and the cultivars experienced different changes facing cold stress. In ‘Perllete’, 4 and 8 h of cold stress increased the total protein content. However, increasing the treatment time up to 16 h led to a significant decrease in the trait. In ‘Khalili’, cold treatments for 4 and 8 h significantly increased the trait, but 16 h was not significantly different from the 8 h treatment. However, in ‘Ghiziluzum’ increasing the cold stress duration led to an upward increase in total protein content. Thus, the lowest total protein content belonged to control treatment of ‘Perllete’ (0.0152 mg/fresh leaf weight), and the highest protein content belonged to ‘Ghiziluzum’ with 16 h of cold treatment (0.616 mg/fresh weight).

Means comparisons (Figure 1C) showed that cold stress caused a significant increase in malondialdehyde content in all studied cultivars compared with the control. With all cultivars, the lowest accumulation of malondialdehyde belonged to the control treatment, and among the cultivars, the lowest value belonged to control of ‘Perllete’ (1.309 nmol/g of fresh weight). The prolonged cold treatment time-course led to an increase in malondialdehyde content in all cultivars; in all cultivars the highest malondialdehyde content belonged to 16 h of cold treatment. Therefore, the highest accumulation of malondialdehyde belonged to 16 h of ‘Khalili’ (2.741 nmol/g of fresh weight). Mean comparisons revealed that, the APX activity in stressed plants was significantly different from control ones (Figure 1D). This difference was not uniform in the cultivars and the different cultivars had diverse reactions to cold stress. Among the cultivars tested, the lowest ascorbate peroxidase activity belonged to the control treatment of ‘Perllete’ (0.209 units of enzyme per min per g of fresh leaf weight) and the highest activity belonged to the 16 h treatment of ‘Perllete’ (2.916 units). Thus, the highest effect on the activity of this enzyme under the influence of cold stress was recorded for ‘Perllete’.

Cold stress significantly increased the content of guaiacol peroxidase in all studied cultivars (Figure 1E). The lowest activity of this enzyme belonged to the control of ‘Khalili’ (2.09 units of enzyme per mg of fresh weight), and the highest amount belonged to ‘Perllete’ with 8 h of cold stress (5.63 enzyme units/mg of fresh weight of leaves). Cold stress caused a significant change in the antioxidant capacity of all studied grape cultivars (Figure 1F). However, this change was not the same in all treatments and cultivars. In ‘Ghiziluzum’, the application of 4 h of stress caused a significant increase in antioxidant activity and reached its maximum with 8 h of cold treatment. However, by increasing the duration of treatment to 16 h there was a decrease in total antioxidant activity in this cultivar. Among the cultivars, the least total antioxidant capacity belonged to the control treatment of ‘Ghiziluzum’ (5.553%), and the highest value belonged to ‘Perllete’ with 16 h of cold treatment (8.309%) and ‘Ghiziluzum’ with 8 h of treatment.

Cold stress increased the accumulation of H_2_O_2_ in the studied grape cultivars (Figure 1G). In ‘Ghiziluzum’, H_2_O_2_ accumulation was significantly increased by cold stress, and increasing the duration of treatment raised the amount of H_2_O_2_ exponentially so that, with 16 h of cold treatment, H_2_O_2_ reached its maximum extent. The lowest accumulation of H_2_O_2_ belonged to the control treatment of ‘Perllete’ (6.34 mmol/L) and, the highest amount belonged to ‘Ghiziluzum’ with 12 h of stress (41.44 mmol/L). Mean comparisons (Figure 1H) showed that cold stress caused a significant increase in electrolyte leakage in all three cultivars compared with control. In all cultivars, the lowest percentage of EC belonged to the control treatment and the highest percentage belonged to 16 h of cold treatment. Moreover, the lowest percentage of EC belonged to the control treatment of ‘Ghiziluzum’ (3.66%), and the highest electrolyte leakage was recorded for ‘Khalili’ with 16 h of cold treatment (17.21%).

### 3.2. Correlation Coefficients

The significant positive correlation was observed among the traits at both 5% and 1% of probability levels in response to cold stress. The significant positive relationship was recorded between the proline content with APX activity and electrolyte leakage. Furthermore, highly positive significant correlation was calculated for total protein and APX activity, H_2_O_2_ and electrolyte leakage. Moreover, the significant positive relationship was observed between GPX with electrolyte leakage. MDA and electrolyte leakage showed positive correlations as well (Table 1).

### 3.3. Principal Component and Cluster Analysis

The principal component analysis (PCA) was exploited to distinguish plot of variation in the physiological attributes and to provide a more applicable understanding of the weight of each characteristic in the total variation. The PCA analysis showed that the three factors or principal components explained 80.46% of total variations (Table 1). The first component (PC1) was the most efficient as it was responsible for about 45.58% of total variance (proline, total soluble protein, APX, GPX and electrolyte leakage) (Table 1 and Table 2). Furthermore, the next two principal components, PC2 (18.13%) and PC3 (16.74%), accounted the rest of total variations (Table 2 and Table 3).

### 3.4. Chlorophyll Fluorescence

**Figure 2:**
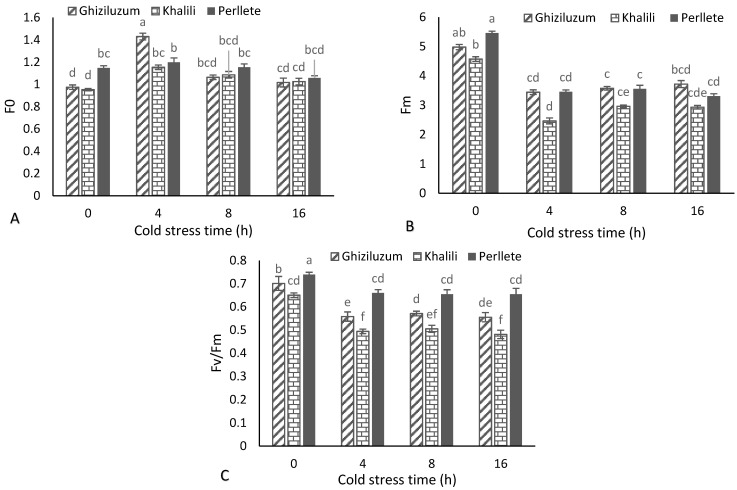
The effects of cold stress time-course on F0 (minimal fluorescence) (**A**), Fm (maximal fluorescence) (**B**) and Fv/Fm (maximum photochemical quantum yield of photosystem II) (**C**) of three grapevine cultivars. Similar letters show no meaningful difference at 5% probability level by Duncan’s Multiple Range Test. Data are mean ± SD (*n* = 3).

One of the methods for estimating the plant damage due to cold stress is chlorophyll fluorescence evaluation. Cold stress increased the minimal fluorescence (F0) in all studied cultivars. In ‘Ghiziluzum’ and ‘Khalili’, 4 h of cold stress significantly increased the minimal fluorescence; however, cold stress for 8 and 16 h caused a decrease in this trait again. Cold stress in ‘Perllete’ did not have a significant effect on this trait and the treatments were not significantly different. The lowest minimal fluorescence belonged to the control treatment of ‘Khalili’ (0.953), and the highest value was belonged to ‘Ghiziluzum’ with 4 h of cold treatment (1.43) (Figure 2A). The mean comparisons depicted (Figure 2B) that cold stress up to 4 h had a negative decreasing effect on maximal fluorescence (Fm) in all studied cultivars, so that the highest maximal fluorescence belonged to the control treatment of ‘Perllete’ (5.46) and the lowest value belonged to ‘Khalili’ with 4 h of cold treatment (2.465). Cold stress in all three cultivars reduced the maximum photochemical quantum yield of photosystem II (Fv/Fm) compared with the control; as in all cultivars, the highest value belonged to the control treatment. The highest amount of Fv/Fm belonged to the control treatment of ‘Perllete’ (0.739) and the lowest amount of Fv/Fm was recorded for ‘Khalili’ with 12 h of cold treatment (0.481) (Figure 2C). At the low temperatures, the metabolism of leaf cells is severely inhibited mainly due to the effect of cold stress on the reduced carbon dioxide fixation or delayed photosynthetic cycles, the changes in the formation, and distribution of sugars, which all reduce the plant’s ability to recover from the stress damages.

## 4. Discussion

In the cold-tolerant plants, proline is the predominant amino acid acting as a protective compound against cold, and the elevated proline concentration in tissues is the mechanism by which plants adapt to withstand cold stress. Moreover, the accumulation of this amino acid has been reported in many plants under cold stress due to the increased biosynthesis or a decreased degradation rate [2,4]. Protecting the plant from osmotic changes, keeping the integrity of biological membranes, maintaining pH, protecting cellular enzymes, as well as storing energy for post-frost recovery are the major functions of proline in face of cold stress [14]. During cold stress, the total protein content increases which goes on to a certain extent and then decreases. These fluctuations can be interpreted that in the onset of stress, the plant begins to increase the expression of genes involved in the biosynthesis of defense enzymes to protect cellular structures to keep their normal activities. Therefore, by producing the sufficient amount of defense enzymes within cells, it is not necessary to further increase the number of enzymes as a subset of the total protein, and behind a sufficient period from the onset of stress, the conditions are under control by the plant cells [2,3,4]. Among the three cultivars studied, the cultivar with the lowest increase in malondialdehyde content compared with the control treatment was ‘Perllete’. Malondialdehyde is one of the end products of membrane lipids peroxidation resulting from the reactive oxygen species activity. In other words, malondialdehyde levels are often used as an indicator of oxidative stress damage [15]. In the present study, cold stress increased the peroxidation of membrane lipids and as a result, increased the amount of malondialdehyde in the tissues of three grape cultivars. Commonly, the level of malondialdehyde in plant tissue is a sign of stress-induced damage. Similar results on the effect of cold stress on malondialdehyde content were obtained in previous studies with different grape cultivars [16]. Based on the results obtained in the present study, ‘Perllete’ can be introduced as the most tolerant among the tested cultivars, and ‘Ghiziluzum’ can be placed in the next rank in terms of cold tolerance. Plants show a variety of morphological, biochemical, and physiological adaptations in response to stresses, including changes in the activities of certain enzymes such as ascorbate peroxidase. According to the literature, ascorbate peroxidase, as the most important antioxidant enzyme in plants, regenerates many free radicals, especially hydrogen peroxide. The importance and role of this enzyme have been emphasized in many other plants especially in tangerine [17]. Higher APX activity was observed in *Jatropha macrocarpa* as a response to high H_2_O_2_, which improved cold stress tolerance. Whereas reduced APX activity in *J. curcas* was linked with the increased sensitivity under cold stress conditions [18]. The results of the study conducted by Karimi Alvije et al. [1] also showed an increase in the content of guaiacol peroxidase in 7 different grapevine cultivars due to cold. They stated that placing grape seedlings at 4 °C initially and significantly increased the content of this enzyme, but then stress caused a decreasing pattern in its content. In cold-tolerant plants, the more efficient mechanisms enable them to protect themselves against the destructive effects of ROSs [4,6]. This group of plants employs enzymes such as superoxide dismutase (SOD), catalase (CAT), ascorbate peroxidase (APX) and glutathione reductase (GR) as well as non-enzymatic compounds including ascorbate, tocopherol, carotenoids and other compounds (including flavonoids, polyanols and mannitol) to gain the ability of escape from the reactive oxygen species damage [1,3,6]. In plant cells, AsA-GSH cycle is the major antioxidant defense pathway to detoxify H_2_O_2_ as a redox homeostasis [17,19]. Hydrogen peroxide and reactive oxygen radicals are produced under natural conditions in tiny quantities during the normal metabolism in diverse organelles, including chloroplasts, mitochondria, peroxisomes and in any places where there is an electron transport chain [5,15]. ‘Ghiziluzum’ with its high H_2_O_2_ production was more sensitive to cold stress than ‘Khalili’ and ‘Perllete’ and had low cold tolerance. Numerous studies, including research on apples and pears, showed an increase in H_2_O_2_ accumulation under stress conditions [20]. Cell membrane is the first site of damage by the cold stress and the changes in membrane state as a result of cold stress causes the membrane malfunction. So, measuring the electrolyte leakage of tissues is an acceptable criterion for evaluating plant resistance to cold stress [17,18]. In the present study, ‘Perllete’ was more tolerant to the cold stress in terms of electrolyte leakage than ‘Khalili’ and ‘Ghiziluzum’. The results obtained were consistent with the findings on different grape cultivars [16] which reported the increased electrolyte leakage in response to the cold stress. A plant’s survival under environmental stresses, especially cold stress, requires the control of harmful compounds biosynthesis inside plant cells, which ensures normal metabolism and fortified survival. These are ROSs and the auxiliary compounds result from the oxidation of biological materials. These compounds cause metabolic disorders due to the intense electron demand. Antioxidant enzymes reduce the cell damage by scavenging ROSs, and antioxidants hamper the oxidation of biomolecules, such as lipid peroxidation, by supplying the electrons needed [4,6,16]. The study revealed that ‘Perllete’ was reasonably low temperature tolerant. Literature review, showed that as reported by Gu et al. [21], *V. amuriensis* was a low temperature tolerant species, similar to our results on ‘Perllete’ the reduced lipids peroxidation and electrolyte leakage were the key factors simulating the stress tolerance. Ascorbate peroxidase activity in the three studied cultivars with the present experiment and, especially in ‘Perllete’ was higher than *V. amuriensis* leading to more efficient H_2_O_2_ scavenging. Moreover, owing to the crucial role of APX in the ascorbate–glutathione cycle, the antioxidant activity was ameliorated facing with low temperatures.

When light is at a moderate level, its majority is employed in photochemical activities for photosynthesis and a small part of its energy is emitted as fluorescence known as basal or minimal fluorescence (F0) [22]. In the present study, the amount of F0 in all studied cultivars increased due to cold application. An increase in F0 indicates damage to the photosystem II electron transfer chain due to a decrease in the capacity of quinone A (QA) and the lack of its complete oxidation to a slow flow of electrons along the photosystem II pathway and the inactivation of photosystem II. A rise in F0 is associated with photoinhibitory damage but not with zeaxanthin retention [23]. The increased F0, due to cold stress has been observed in plants such as basil (*Ocimum basilicum* L.) and lettuce (*Lactuca sativa*) [24]. The stress conditions cause structural changes in the pigments in photosystem II and the fluorescence function such as maximal fluorescence is changed, making it possible to use these factors as an indicator for estimating stress-induced damage to the plant photosynthetic system [22,23]. The results showed that cold reduced the maximal fluorescence in all studied cultivars and this drop of Fm occurred at the maximum by applying 4 h of cold stress. Researchers suggested that a decrease in Fm may be related to a decrease in the activity of the water-degrading enzyme complex as well as the electron transfer cycle in/or around photosystem II [23,24]. Research on tomatoes (*Solanum lycopersicum*) [24], also showed a decrease in Fm due to cold stress. Therefore, the measurement of Fv/Fm can be used as a successful method to determine the status of photosynthetic apparatus and identify the degree of cold tolerance in plants [22]. By slowing down the insertion of protein D1 into the center of photosystem II, cold stress slows down the plants recovery and causes membrane degradation and chlorophyll oxidation, thereby reducing the Fv/Fm ratio (maximum photochemical quantum yield of photosystem II under adaptive conditions to light); it is an estimate of the maximum photochemical quantum yield of photosystem II [25]. In many plant species, when Fv/Fm ratio is about 0.7 to 0.8, it means that no stress has been applied on the plant. Therefore, values less the abovementioned indicate the effects of stress on plants [22,23,24]. In fact, chlorophyll fluorescence indicates a decrease in the initial health of the plant before the signs of deterioration; this trait indirectly indicates health (fluidity, stability, and cohesion) of photosynthetic membranes [22,25]. Considering that high Fv/Fm indicates high cold resistance, among the three studied grapevine cultivars, ‘Perllete’, which has the highest maximum photochemical quantum yield of photosystem II facing cold, was more cold-tolerant than the other two cultivars. Cold stress increases F0 and decreases Fv/Fm, which indicates the discontinuity of light-absorbing pigments from the photosystem II complex, leading to a decrease in the quantum performance of photosystem II. The activity of the photosystem II is severely reduced or even stopped under cold conditions, and chloroplasts, stromal carbon metabolism, and photochemical reactions in the thylakoid lamella have been cited as primary sites of cold stress injury [22,23,24,25].

## 5. Conclusions

In all three cultivars, the activities of guaiacol peroxidase and ascorbate peroxidase enzymes and total antioxidant capacity were increased in response to cold exposure. Furthermore, cold stress exposure increased the accumulation of proline in leaf tissue in all cultivars. Electrolyte leakage and the concentrations of malondialdehyde and hydrogen peroxide, as signs of cold damage, increased; however, this increase was different in various cultivars and cold levels. Cold stress caused damage to the photosynthetic system and therefore increased minimal fluorescence and decreased the maximal fluorescence and maximum photochemical quantum yield of photosystem II. This damage was less in ‘Perllete’ than the other two cultivars. Since early spring low temperatures incidence is a limiting factor in vineyards development in major parts of Iran and many other countries, tracing the related biomolecules such as membrane integrity indices and chlorophyll fluorescence can be reliable criteria in deciding on the biochemical variations at the cellular level and the photochemical efficacy under stressful environments.

## Figures and Tables

**Table 1:** Correlation coefficient values for the pairs of studied characters in three grapevine cultivars under cold stress.

Correlation Coefficient Values								
	Y1	Y2	Y3	Y4	Y5	Y6	Y7	Y8
Y1	1							
Y2	0.195	1						
Y3	0.748 **	0.562 **	1					
Y4	0.266	0.307	0.476 **	1				
Y5	0.108	0.792 **	0.406 *	0.274	1			
Y6	0.190	0.339*	0.043	−0.090	0.287	1		
Y7	0.021	0.147	0.101	0.294	0.112	−0.323	1	
Y8	0.536 **	0.508 **	0.409 *	0.217	0.235	0.598 **	−0.181	1

**, *, significant at *p* ≤ 0.01 probability and significant at *p* ≤ 0.05 probability, respectively. **Y1** (Proline), **Y2** (Protein), **Y3** (APX), **Y4** (Total antioxidant), **Y5** (H_2_O_2_), **Y6** (MDA), **Y7** (GPX), **Y8** (Electrolyte leakage).

**Table 2:** Total variance explained.

Component	Initial Eigenvalues % of Cumulative		
	Total	Variance	%
1	3.647	45.589	45.589
2	1.450	18.131	63.720
3	1.340	16.746	80.466
4	0.736	9.194	89.660
5	0.336	4.202	93.862
6	0.297	3.717	97.579
7	0.139	1.738	99.317
8	0.055	0.683	100.000

Extraction Method: Principal Component Analysis.

**Table 3:** Component matrix.

Component			
	1	2	3
Y1	0.754	−0.599	0.007
Y2	0.746	0.562	−0.097
Y3	0.820	−0.206	−0.379
Y4	0.438	0.063	−0.643
Y5	0.600	0.653	−0.176
Y6	0.443	0.303	0.732
Y7	0.728	−0.460	0.166
Y8	0.755	−0.023	0.423

Extraction Method: Principal Component Analysis; 1 (principal component 1), 2 (principal component 2) and 3 (principal component 3). Three components extracted. Y1 (Proline), Y2 (Total soluble protein), Y3 (APX), Y4 (Total antioxidant), Y5 (H2O2), Y6 (MDA), Y7 (GPX), Y8 (Electrolyte leakage).

## Data Availability

All new research data were presented in this contribution.
